# From dispersal to predation: A global synthesis of ant–seed interactions

**DOI:** 10.1002/ece3.4377

**Published:** 2018-07-30

**Authors:** Hannah J. Penn, Thomas O. Crist

**Affiliations:** ^1^ Department of Entomology Louisiana State University Baton Rouge Louisiana; ^2^ Department of Biology Miami University Oxford Ohio

**Keywords:** ecosystem services, Formicidae, parasitism, seed fate

## Abstract

Ant–seed interactions take several forms, including dispersal, predation, and parasitism, whereby ants consume seed appendages without dispersal of seeds. We hypothesized that these interaction outcomes could be predicted by ant and plant traits and habitat, with outcomes falling along a gradient of cost and benefit to the plant. To test this hypothesis, we conducted a global literature review and classified over 6,000 pairs of ant–seed interactions from 753 studies across six continents. Linear models showed that seed and ant size, habitat, and dispersal syndrome were the most consistent predictors. Predation was less likely than parasitism and seed dispersal among myrmecochorous plants. A classification tree of the predicted outcomes from linear models revealed that dispersal and predation formed distinct categories based on habitat, ant size, and dispersal mode, with parasitism outcomes forming a distinct subgroup of predation based on seed size and shape. Multiple correspondence analysis indicated some combinations of ant genera and plant families were strongly associated with particular outcomes, whereas other ant–seed combinations were much more variable. Taken together, these results demonstrate that ant and plant traits are important overall predictors of potential seed fates in different habitat types.

## 1 INTRODUCTION

Consumer–resource and mutualistic species interactions are known to vary from mutualism to antagonism depending on the specificity and context of the interaction or spatial variation in environmental conditions (Bronstein, 2015; Bronstein, Wilson, & Morris, 2003). Mycorrhizal associations between plants and fungi, for example, can result in mutualistic or pathogenic outcomes to plant fitness, depending on the mycotrophic status of the plant and local adaptation of mycorrhizal fungi (Brundrett, 2004; Johnson, Graham, & Smith, 1997). Likewise, plant–animal interactions such as pollination or frugivory range from mutualistic to antagonistic depending on the behavioral responses of animals to floral or fruit traits of plants (Case & Bradford, 2009; Ramsey, 1988; Tyre & Addicott, 1993; Whitehead & Poveda, 2011). Flower‐visiting insects and birds may specialize on a small number of flowering plant species resulting in efficient pollen transfer between conspecific plants, whereas others may be inefficient at pollen transfer or become parasitic by robbing nectar without pollination (Allsopp, de Lange, & Veldtman, 2008; Mauck & Burns, 2009; Smithson, 2009). Similarly, seed predators may act as seed dispersal agents of uneaten seeds due to caching behavior or the movements of seeds to safe sites for germination (Detrain & Tasse, 2000; Vander Wall, Kuhn, & Gworek, 2005).

Plants often form coevolved relationships with ants that may manifest on a gradient of mutualism to antagonism (Bronstein, 1994; Bronstein et al., 2003; Davidson & Morton, 1981; Del Val & Dirzo, 2004). High‐fidelity mutualistic myrmecophytes like Acacias provide both housing and food to ants in return for physical protection from insect and vertebrate herbivory (Heil, Baumann, Krüger, & Linsenmair, 2004; Janzen, 1966; Palmer et al., 2008) or release from plant–plant competition (Fiala, Maschwitz, Pong, & Helbig, 1989; Janzen, 1969). Many plant species do not exhibit quite this level of commitment but will offer a simple food reward in the form of lipid‐rich elaiosomes or Beltian bodies and nectar from extrafloral nectaries and flowers (Dutton, Shore, & Frederickson, 2016; Fischer, Richter, Hadacek, & Mayer, 2008; Heil et al., 2001; Rickson, 1975). These plant traits are meant to elicit a positive ant response to further plant protection or fitness (Gorb & Gorb, 1995; Imbert, 2006), but, in fact, may result in parasitism similar to those found in pollination and mycorrhizal associations (Andersen & Morrison, 1998; Aranda‐Rickert & Fracchia, 2011). The antagonistic extreme of this gradient comprises direct and indirect consumption of plant resources to the detriment of plant fitness (Brown & Davidson, 1979; LeVan & Holway, 2015; Schultz et al., 2015). For instance, ants might farm and spread honeydew‐producing hemipterans, which both consume the plant and vector diseases, in lieu of plant‐mediated nectar rewards (Hawkes & Jones, 2005; Offenberg, 2001).

Ant‐mediated seed dispersal, or myrmecochory, is well studied and encapsulates the varied gradient of species interactions incentivized by food rewards (Beattie, 1985; Pizo & Oliveira, 2001; Timoteo, Ramos, Vaughan, & Memmott, 2016). The benefit to the plant is that seeds can be dispersed well away from parent plants, as documented in temperate forest systems (Andersen, 1988; Takahashi & Itino, 2012). In post‐seed dispersal by ants, seeds are removed from predation risk posed by other granivores such as rodents and ground beetles, increasing plant fitness (Heithaus, 1981; Vander Wall, Kuhn, & Beck, 2005). Furthermore, if the seed is taken into an ant nest for elaiosome removal, it is shielded from damaging abiotic conditions such as fire and drought (Bebawi & Campbell, 2002; Bebawi, Campbell, & Mayer, 2012). In exchange for these services, participating plants often provide fruit or an elaiosome, a lipid‐rich appendage that is easily removed and consumed after dispersal events and has been shown to increase ant fitness (Ciccarelli, Andreucci, Pagni, & Garbari, 2005; Gammans, Bullock, & Schonrogge, 2005; Garrido, Rey, & Herrera, 2009; Lengyel, Gove, Latimer, Majer, & Dunn, 2010). However, parasitic ant species consume the fruit or elaiosomes in situ, thereby removing the incentive for future dispersal of those seeds (Beaumont, Mackay, & Whalen, 2011; Christianini, Mayhe‐Nunes, & Oliveira, 2012; Guimaraes & Cogni, 2002). Ant predation of seeds (granivory) can be regarded as the antagonistic end of the myrmecochory spectrum as these ants destroy seeds, reducing plant fitness (MacMahon, Mull, & Crist, 2000; Plowes, Johnson, & Hoelldobler, 2013; Rissing, 1981). Granivorous ants like those in the genera *Pogonomyrmex* and *Messor* use seeds as their primary food source and actively remove plants near their nests to the detriment of their preferred seed suppliers (Belchior, Del‐Claro, & Oliveira, 2012; Clark & Comanor, 1975; Crist & MacMahon, 1992). The impacts of the selective foraging conducted by these ants have the potential to greatly change the underlying seed bank and resulting vegetative structure within their foraging ranges (Andrew, 1986; Azcarate & Peco, 2006; Briggs & Redak, 2016; Brown & Human, 1997; Clark & Comanor, 1975).

**Figure 1:**
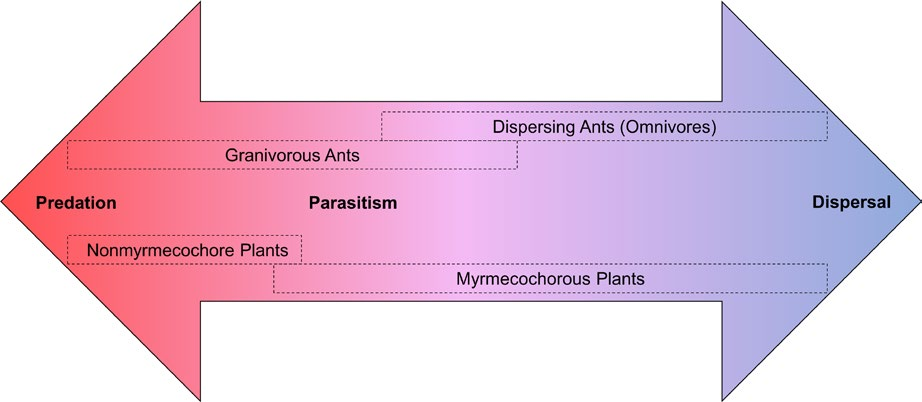
Predicted relationship of select ant and plant traits with ant–seed interaction outcome. The *x*‐axis represents a theoretical gradient of benefits/costs to the plants with the placement of ant and plant traits related to seed outcomes based on hypothesized probability occurrence

We can visualize these ant–seed interactions along a spectrum of cost and benefit to the plant (Figure 1). At one end of the spectrum are ant mutualists that provide the greatest benefit to the plant by dispersing but not damaging seeds; at the other end are ants that harm the seed to the point of destroying its opportunity to germinate. As in other plant‐based systems, some behaviors may not be entirely classified as myrmecochory or granivory and are fraught with nuance. Granivorous ants are often assumed to consume seeds without providing any dispersal (Anderson & MacMahon, 2001; Predavec, 1997; Saba & Toyos, 2003), but may actually increase germination success by moving seeds meant for consumption to favorable microclimates in their nests (Brown, Scherber, Ramos, & Ebrahim, 2012; Dean & Yeaton, 1992). Alternatively, dispersing ant species may move seeds into their nests where the seeds are then too deep to germinate, thus removing the benefit to the plant (Bas, Oliveras, & Gomez, 2007; Christianini, Mayhe‐Nunes, & Oliveira, 2007; Renard, Schatz, & McKey, 2010). Parasitic behaviors may also not be straightforward, as elaiosome removal may increase the likelihood of seed germination if ant cleaning confers fungal protection (Ohkawara & Akino, 2005). Even seeds not actively participating in myrmecochory can receive a germination boost from parasitic ants that scar or crack the seed coat (Leal & Oliveira, 1998; Oliveira, Galetti, Pedroni, & Morellato, 1995). The interacting ant and plant species and their associated traits may dictate the particular placement of such complex ant–seed interactions along the gradient of mutualism and antagonism.

Estimating where particular ant–plant interactions fall along this gradient would allow for future predictions and management decisions. Granivory is often of concern when the plant is endangered (Albert, Escudero, & Iriondo, 2005), is an invasive (Alba‐Lynn & Henk, 2010), or is in agricultural/pasture setting where sown seeds are consumed (Baraibar, Ledesma, Royo‐Esnal, & Westerman, 2011; Campbell & Campbell, 1966; Campbell & Swain, 1973; Diaz, 1992). Prior knowledge of how ants and seeds interact has been applied for the protection of endangered plant species such as increasing plant distributions via ant dispersal or by protecting those plants from ant predation (Boyd, 2001; Cursach & Rita, 2012; Fisher et al., 2014). Similarly, within agroecosystems, ants may contribute to nonchemical weed control where herbicide resistance is problematic (Baraibar, Westerman, & Recasens, 2009; Comas, Royo‐Esnal, Recasens, & Torra, 2016; Evans & Gleeson, 2016; Jacob, Minkey, Gallagher, & Borger, 2006; Seaman & Marino, 2003; Westerman, Atanackovic, Royo‐Esnal, & Torra, 2012). If traits of ants and plants are associated with a likely seed fate, then we could attempt to predict the outcomes of ant–seed interactions in similar situations but in dissimilar geographic areas or habitats (Poff, 1997; Pyšek et al., 2012; Thuiller, Richardson, Rouget, Procheş, & Wilson, 2006).

The aim of this study was to review past literature on ant–seed relationships to determine the probability of the outcomes between ant–seed interactions (dispersal, predation, and parasitism) based on the identity and characteristics of the corresponding ants and plants. We hypothesized that the outcome of ant–seed interactions could be predicted by ant and plant traits as well as habitat type and continent of origin. Based on previous work, we predicted that ant species classified as granivores would be closely associated with seed predation, particularly with grasses in arid environments like deserts. Omnivorous ant species (i.e., not known granivores or mutualists) were expected to have less association with any particular interaction outcome as these ants may interact with seeds based on habitat context rather than dietary specialization. Furthermore, seed predation studies have shown that seed size and shape are important predictors of predation, but ant body size or ant–seed size matching may also be important to predation or dispersal. To this end, we expected that more ergonomic seeds (i.e., long, thin seeds or those with a “handle”) would have a smaller likelihood of parasitism versus seeds that are difficult to carry for either predation or dispersal purposes. In the same vein, we proposed that larger ants with an increased ability to carry seeds will be less like to be parasitic compared to small ants such as thief ants. We also expected that the habitat or context of the interaction would influence the outcomes of ant–seed interactions. For instance, interactions involving more cosmopolitan ants may differ among the habitats given that these areas provide different resources and sources of competition. Finally, we expected that the observed interaction outcomes would fall along a gradient of cost and benefit to the plant with dispersal (mutualism) and predation (antagonism) located at opposite extremes, and with parasitism (destruction of attached seed coat, fruit, or elaiosome without seed dispersal or consumption) located near predation as it presumably harms the seed. Alternatively, parasitism may be more closely associated with dispersal as the seed components meant to aid in dispersal may also lead to parasitism.

## 2 MATERIALS AND METHODS

### 2.1 Data collection and preparation

The initial literature search was conducted during January 2017 in the ISI Web of Science search engine (Giladi, 2006) using the search terms “ant” and “seed” for topic with no restrictions on year on publication. Relevant literature cited in included papers and related review papers were added to the list of references to be analyzed for inclusion. For data collection, review papers and conferences proceedings were excluded as were references that did not directly observe an explicit ant–seed interaction (Supporting Information Tables S1 and S2). Each reference was reviewed for information on plant species, if the plant was myrmecochorous, ant species, habitat, country of observation, and ant–seed interaction type (dispersal, predation, and parasitism/consuming or removing of seed coat, fruits, or elaiosomes attached to the seed in situ). All variables collected for coded as categorical. Each unique plant and ant combination per reference was considered an observation and may have included multiple outcomes for that plant and ant species combination; therefore, interactions were coded in three separate columns.

After all references were reviewed for appropriate data, corresponding plant and seed traits were obtained through a search of online databases (Blittersdoriff, Dressler, Schmidt, & Zizka, 2011; Danin & Fragman‐Sapir, 2017; “eFloras,” 2017, “Encyclopedia of Life,”^2017^, “SEINet – Arizona Chapter Image Library,” 2017, “The plant list v.1.1,” 2017, 2017, “World wide wattle v.2,” 2017; Kew Royal Botanic Gardens, 2017; Michail Belov, 2017; South Australian Seed Conservation Centre, 2017; USDA, 2017). Characteristics included updated plant family, growth habit (forb, woody, grass, other which was typically cactus and bromeliads, and forb/woody for species that may do either but were not specified), life‐form (annual/biennial, perennial, and both or those that might be either one but were not specified), primary dispersal mechanism (auto‐, anemo‐, zoo‐, and myrmecochory), seed length (mm), and seed shape (long/narrow, sphere, round/flat, and odd). We note that many of the plant diaspores in this review are actually fruits, but we refer to them as seeds for simplicity. Ant genus and species names were updated on 9 May 2017, and an approximation of ant length was obtained via the mean of scaled photographs of 1–3 (as available) randomly selected specimens of minor workers for each species (AntWeb v.6.61, 2017). Ant feeding habits were obtained from AntWiki and AntWeb (AntWeb v.6.61, 2017, AntWiki, 2017) and coded as plant consumers (fungus‐farmers and granivores), honeydew consumers (typically farms aphids or scales), omnivores (any species with more than one listed habit or those listed solely as omnivores and scavengers), or other (predators, parasites on other ants, and unknown). The continent (categorical) was also for each observation added based on the manuscript‐provided GPS coordinates or country of observation data.

Data were cleaned by recategorizing the stated habitat type into forest, desert, grassland, or human (including both agriculture and urban environments) (IUCN, 2017) and by converting country of origin to continent as many countries were associated with fewer than ten observations. Seed length (mm) and ant length (mm) were transformed as follows (where “x” represents length): size = log_10_(*x*). Observations missing any of the above variable information were excluded from final analyses. The final dataset used for all analyses is included in Supporting Information Table S3 with a legend of terms in Supporting Information Table S4.

### 2.2 General linear models to determine impact of traits and environment on seed outcomes

All analyses were completed using R v3.2.3 (R Foundation for Statistical Computing, 2017). Three generalized linear models were constructed separately to predict the probability of seed dispersal, predation, and parasitism using the glmer function and the binomial family and logit link responses in the lme4 package (Bates et al., 2017). Continent was included as a random effect in all models. The fixed effects were screened (Supporting Information Table S5) using forward then backward variable selection using a combination of Akaike's Information Criterion (AIC) and the Bayesian Information Criterion (BIC). Variables would be included as fixed effects if AIC was reduced by >4 and did not increase BIC >4 (Ockinger et al., 2010). Variables suspected of association were tested for independence using Pearson's chi‐square test with a Yates continuity correction before model selection. If there were significant associations between pairs of variables, the variable with the most biological relevance was selected. For example, information on myrmecochory gathered from online databases was used in lieu of information gleaned directly from the papers as they were correlated and the database data were more consistent. The following variable sets were screened as fixed effects in the same order for each linear model—seed length, ant length (hereafter seed and ant size, respectively), habitat, dispersal mechanism, ant diet, plant life‐form, plant growth habit, and seed shape. Categorical variables were tested alternatively as either one factor containing all levels of the factor or with each factor level as individual dummy variables. Two‐way interactions between variables were also screened, but higher order interactions were ignored. Post hoc Tukey tests were conducted to detect differences in observed outcome probabilities among the habitat categories as well as those of seed shape (results listed in the Supporting Information Table S6).

### 2.3 Classification tree of interaction outcomes

To synthesize the results of the three sets of general linear models, we used the predicted probabilities for dispersal, predation, and parasitism to classify the interaction outcome of each observation based on the largest of the predicted probabilities from the three general linear models. The three‐response categorical variable (dispersal, predation or parasitism) was then used to construct a classification tree with the same eight predictor variables that were common to the three sets of general linear models. The classification tree was created and plotted using recursive partitioning with the rpart and rpart.plot functions of the R programming language (Milborrow, 2017; Therneau, Atkinson, & Ripley, 2018). All eight predictor variables were maintained in the final classification tree with each having a complexity parameter >0.01.

### 2.4 MCA for estimation of interaction gradients and taxonomic associations

To test the roles of plant and ant taxa in determining the outcomes of ant–seed interactions, we conducted multiple correspondence analysis (MCA) using the MCA function in the R package FactoMineR (Husson, Josse, & Le, 2008; Husson, Josse, Le, & Mazet, 2017). The MCA used the plant family, ant genus, and seed fate (dispersal, predation, parasitism) of each observation to determine if particular subsets of interactions between ant and plant taxa were associated with seed fates.

## 3 RESULTS

### 3.1 Data description

Key term searches and cited references yielded 1,844 peer‐reviewed manuscripts of which 753 were appropriate for inclusion. From the included studies, 6,164 unique combinations of interactions between ants and seeds were recorded as binary (0 or 1) categorical outcomes of dispersal, predation, or parasitism (Table 1) and used in the statistical analysis. The most common plant families observed included Poaceae (*n* = 1,791), Fabaceae (*n* = 810), and Euphorbiaceae (*n* = 367) with the most common species being *Ornithopus compressus* L. (Fabaceae, *n* = 152), *Oryza sativa* L. (Poaceae, *n* = 106), and *Helleborus foetidus* L. (Ranunculaceae, *n* = 93). Just under 100 ant genera were recorded with *Messor* (*n* = 2,371), *Pheidole* (*n* = 892), *Solenopsis* (*n* = 339), and *Pogonomyrmex* (*n* = 282) the most commonly observed. We recorded 399 ant species, with *Messor barbarus* L. the most common (*n* = 716) followed by *Messor capitatus* Latreille (*n* = 531), and *Messor hispanicus* Santschi (*n* = 393). The countries of Portugal (*n* = 1,934) and Brazil (*n* = 1,734) had the greatest number of unique observations, which was also reflected within continents (Europe = 2,475; South America = 2,048).

**Table 1:** Number of observations by seed outcome. Some observations encompassed multiple seed outcomes

	Dispersal	Predation	Parasitism	Total
No. Observations	3,703	1,960	507	6,164
Habitat	5	5	5	5
Country	33	39	12	47
Continent	6	6	6	6
Plant family	92	102	45	134
Plant species	399	560	88	863
Ant genera	83	62	52	97
Ant species	255	159	81	399

### 3.2 General linear models of ant–seed interaction outcomes

**Figure 2:**
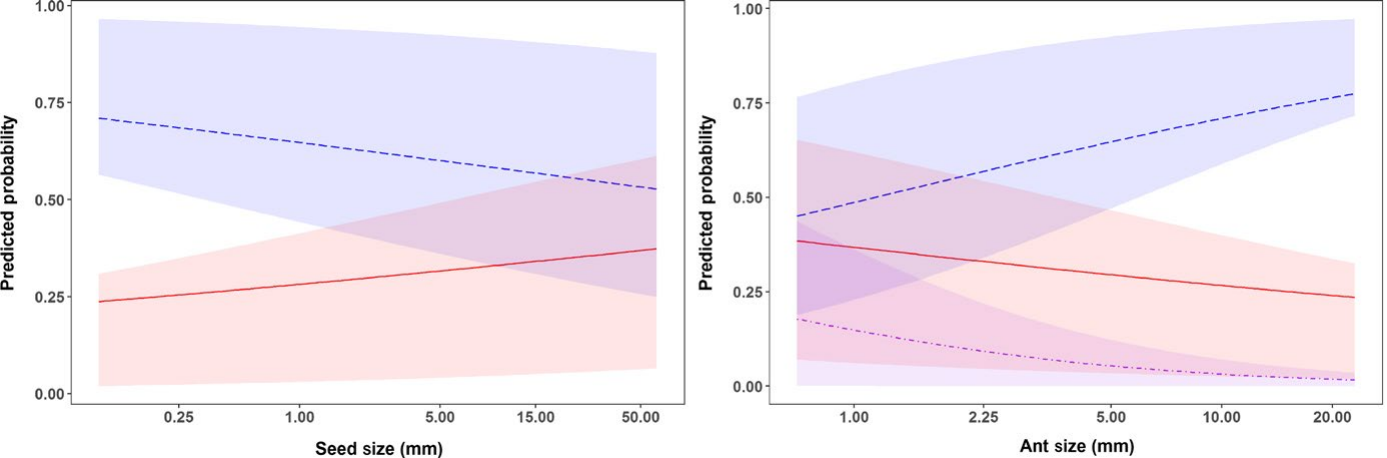
Predicted probabilities of ant–seed interaction outcomes from generalized linear models of dispersal (blue, dashed line), predation (red, solid line), or parasitism (purple, dot‐dashed line) given average seed size (left) and ant size (right). Lines indicate mean predicted probabilities; shaded areas indicate the interquartile range (25%–75%) of the prediction intervals using both the fixed and random effects for the best‐fitting models

**Figure 3:**
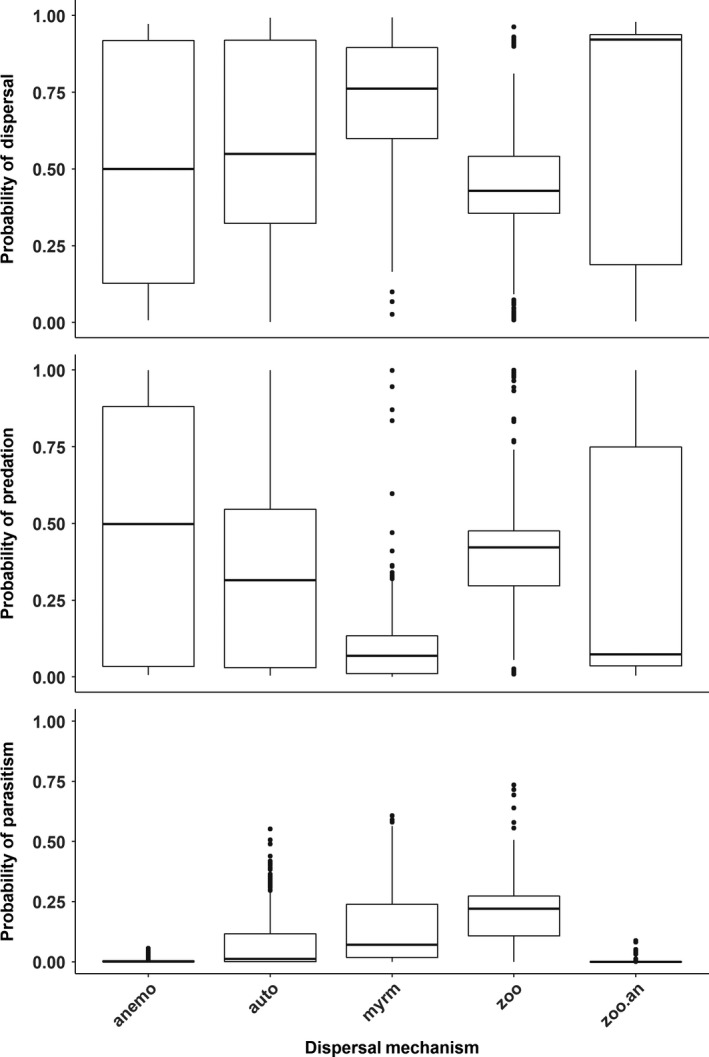
Predicted probabilities of ant–seed interaction outcomes from generalized linear models of dispersal, predation, or parasitism according to the plant's primary dispersal mechanism. Horizontal lines are means, boxes are interquartile ranges, and whiskers are 95% prediction intervals including both fixed and random effects in the models. Anemo = wind; auto = self, myrm = ant; and zoo = vertebrate dispersal; zoo.an = combination wind and vertebrate‐dispersed

**Figure 4:**
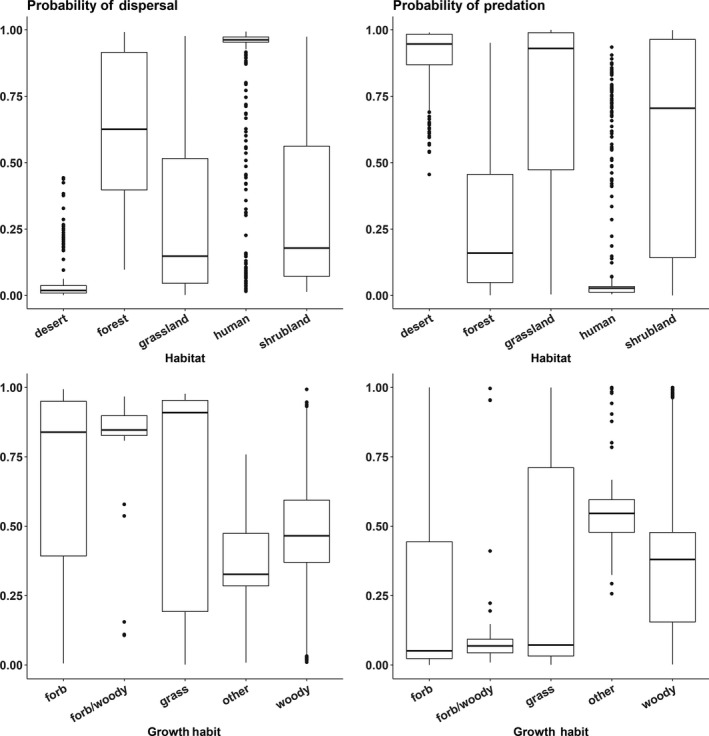
Predicted probabilities of ant–seed interaction outcomes from generalized linear models of dispersal or predation by habitat type and plant growth form. Horizontal lines are means, boxes are interquartile ranges, and whiskers are 95% prediction intervals including both fixed and random effects in the models

Several variables were included in the best‐fitting models of the seed outcomes—seed size, ant size, myrmecochory, if the growth habit was forb/herb, whether or not ants were known seed/plant consumers, and seed shape (Table 2). Larger seed size favored predation but lowered probabilities of dispersal, while increased ant size increased dispersal but decreased predation (Figure 2). Ant size played a role in parasitism where smaller ants had a higher probability of parasitism. Growth habit had a very significant impact on all three seed outcome models with grasses and other types being the reference (Figure 3). Forbs were positively associated with dispersal but negatively so with predation and parasitism, while woody plants were negatively associated with only dispersal and predation. Growth habit, particularly forb and woody plants, reflected a similar trend with myrmecochorous dispersal—these growth habits increased the likelihood of dispersal but not that of predation. Non‐ant‐mediated plant dispersal mechanisms were generally not included in the models and considered the reference for dispersal mechanism (Figure 4). In all models, the seed shape long/narrow was considered the reference type and all other types responded similarly within each respective model. All other seed types were negatively associated with dispersal and parasitism but positively associated with predation.

**Table 2:** Model outcomes (log odds) for with seed dispersal, predation, and parasitism by ants but not including interaction effects (Supporting Information Table S7). Variables without values were not included in the final model. Continent was included as a random effect in all models. For models with more than one subcategory included, the reference category is “forest” for the variable “habitat,” while the reference is “long/narrow” for the variable “shape” and “grass” for the variable “growth habit”

Variable	Type	Dispersal			Predation			Parasitism		
		Estimate	*Z*‐value	*p*‐Value	Estimate	*Z*‐value	*p*‐Value	Estimate	*Z*‐value	*p*‐Value
Intercept		−0.587	−1.366	0.172	0.439	0.661	0.508	−0.674	−1.409	0.159
Seed size		−0.489	−4.648	0.000	0.455	4.228	0.000			
Ant size		1.572	10.090	0.000	−0.897	−4.951	0.000	−2.005	−8.608	0.000
Forest	Habitat									
Desert	Habitat	−3.786	−5.128	0.000	0.978	3.042	0.002	0.364	1.839	0.066
Grassland	Habitat	−1.086	−4.802	0.000	0.751	2.636	0.008			
Human	Habitat	−1.915	−4.215	0.000	−0.043	−0.113	0.910			
Shrubland	Habitat	−0.281	−1.309	0.191	0.042	0.192	0.848			
Myrmecochory	Dispersal mechanism	0.642	5.148	0.000	−1.675	−12.243	0.000			
Anemochory	Dispersal mechanism							0.926	7.064	0.000
Granivory	Ant diet	0.507	3.434	0.001	0.460	4.018	0.000	−2.723	−5.433	0.000
Honeydew	Ant diet				−0.805	−4.761	0.000	0.507	3.542	0.000
Omnivory	Ant diet				−0.178	−1.402	0.161			
Perennial	Life‐form	0.426	3.798	0.000	−0.921	−7.101	0.000			
Annual	Life‐form									
Forb	Growth habit	0.022	0.082	0.935	−1.248	−8.885	0.000	−3.950	−3.931	0.000
Woody	Growth habit	−0.296	−0.970	0.332	−0.722	−6.301	0.000	−0.931	−5.249	0.000
Odd	Seed shape	−0.932	−2.910	0.004	0.763	4.418	0.000			
Round	Seed shape	−0.926	−5.550	0.000	0.789	5.742	0.000	−2.037	−6.637	0.000
Sphere	Seed shape	−1.274	−4.803	0.000	0.061	0.359	0.720	−1.483	−5.789	0.000

**Figure 5:**
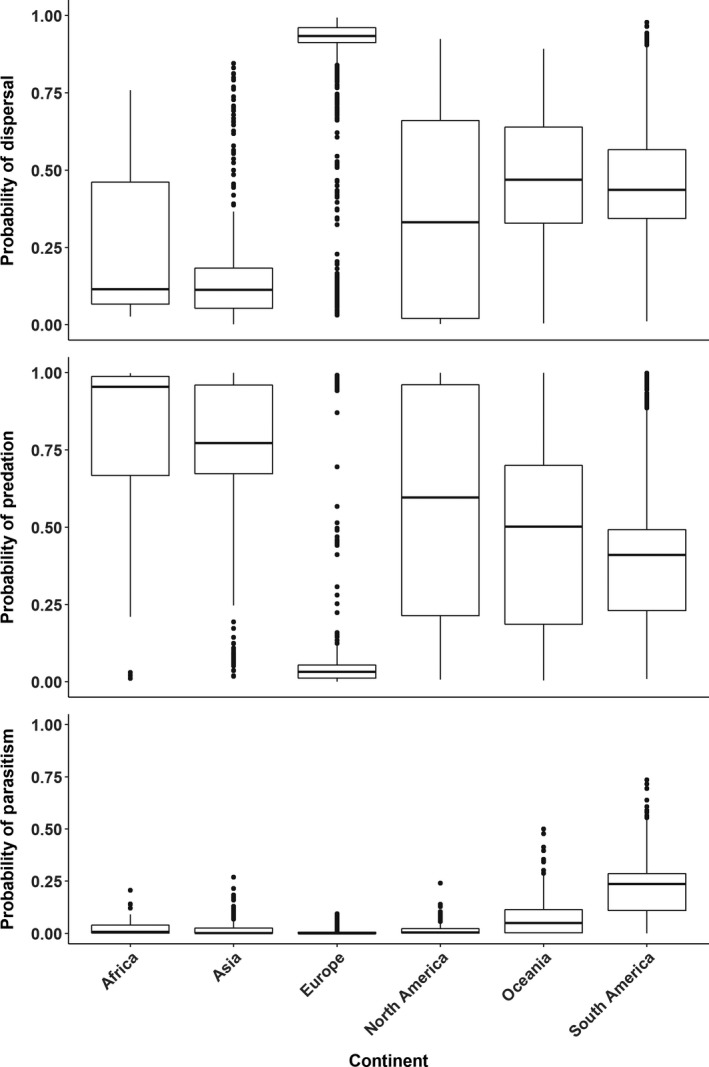
Predicted probabilities of ant–seed interaction outcomes of dispersal, predation, or parasitism by the random effect of continent (i.e., study location). Horizontal lines are means, boxes are interquartile ranges, and whiskers are 95% prediction intervals including both the fixed and random effects in the models

Outside of the above variables, models for dispersal and predation had the most variables in common but with opposite correlations (Table 2). The most intuitive of these—the observed ant species being a known plant consumer—indicated that plant consumers were positively associated with seed predation but negatively associated with dispersal as compared to ants not listed as consuming plants (both honeydew consumption and omnivory). Plant and seed characteristics such as life‐form were important model predictors but were more variable in their impacts. Perennial plants in comparison with annuals/biennials were positively associated with dispersal but negatively with predation. Similar to dispersal, the parasitism model indicated a negative association with annuals/biennials in comparison with perennials, which might indicate a perennial correlation with myrmecochory. Long and narrow seeds, often associated with grasses, were also more likely to be dispersed than predated, whereas other seed shapes had very similar probabilities of dispersal and predation events. The habitat where the ant–seed interaction was observed also had varying impacts on dispersal and predation (Figure 3). Forest and human‐mediated habitats had the greatest probabilities of dispersal, while desert, grassland, and shrubland had the greatest probabilities of predation. For all models, the coefficients for the random effect of continent were observed to change per seed outcome (Figure 5).

### 3.3 Classification tree of interaction outcomes from general linear models

**Figure 6:**
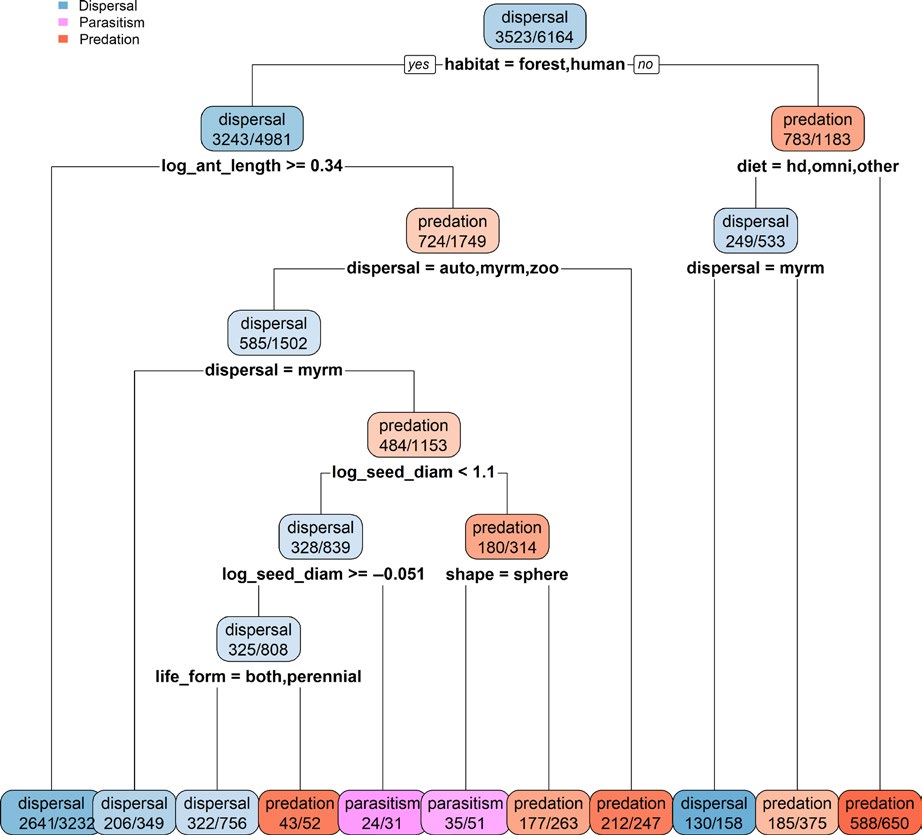
Classification tree for probabilities of dispersal, predation, and parasitism based on the composite set of predictor variables in Table 1. Values in each oval are the number of predicted outcomes (numerator) and the total number of observations (denominator). Abbreviations: habitat = habitat type (forest, human‐dominated, desert, grassland, shrubland); diet = ant diet (hd = honeydew; omni = omnivore; other = predator/parasite); dispersal = primary plant dispersal mechanism; life_form = plant life‐form (both = can be considered both an annual and perennial); shape = seed shape

The classification tree of the predicted outcomes from linear models revealed that dispersal and predation formed distinct categories based on habitat, ant size, and dispersal mode, with parasitism outcomes forming a distinct subgroup of predation based on seed size and shape (Figure 6). Dispersal was the most commonly predicted outcome (3,302 or 55% of the 5,959 observations) from ant–seed interactions in forest and human‐dominated habitats and those involving for larger ants (>2 mm), perennial plants, and plants with an a priori dispersal classification as myrmecochores. However, seed dispersal mode was useful as a predictor of interaction outcome in grasslands and deserts where predation was more common (Figure 6). Predation (1206 outcomes or 20% of total) was most commonly predicted in desert, grassland and shrubland habitats, or among smaller ants (<2 mm) and larger seeds with myrmecochorous or zoochorous dispersal modes (Figure 6). Parasitism was rarely predicted as the highest probable outcome (only 35 or <1%) but was most common with small ants, and large seeds that were more spherical in shape (Figure 6), both making it more difficult for ants to carry the seed rather than to consume the accessory structure in place. A total of 1,416 outcomes (24%) were undetermined based on the eight predictor variables or where the three interaction outcomes occurred with similar probabilities.

### 3.4 Multiple correspondence analysis

**Figure 7:**
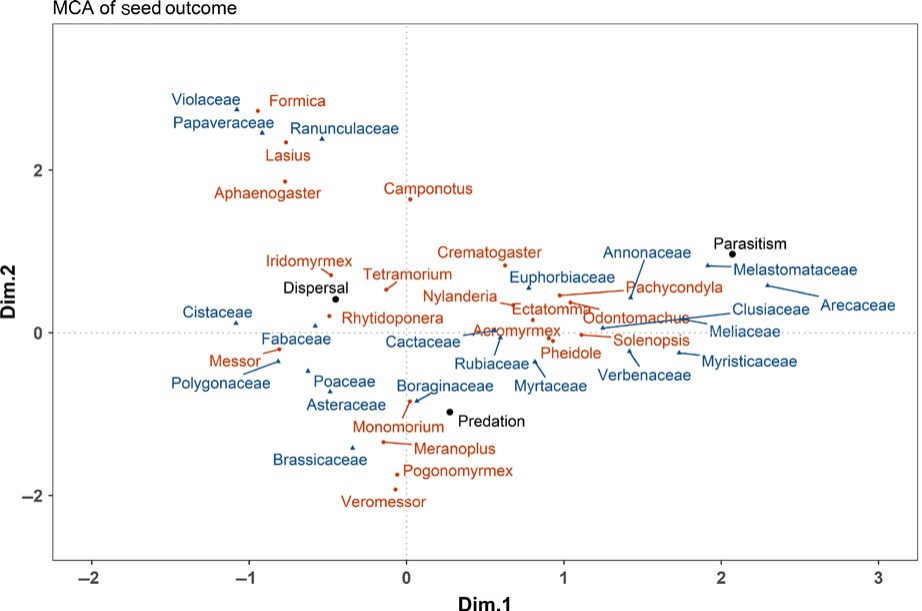
Multiple correspondence analysis of ant–seed interaction outcome (black text and circles) with ant genera (red text and circles) and plant families (blue text and triangles) with only genera/families with >50 observations shown

The multiple correspondence analysis indicated that ant genera and plant families were important predictors of seed outcome. The outcome represented by the first two principal axes corresponding to the greatest eigenvalues (Supporting Information Table S8)—one from dispersal to predation on Dimension 2 and the other from nonparasitism to parasitism on Dimension 1 (Figure 7). The MCA loadings for these axes by general variable category included seed outcome (Dim.1 = 0.538, Dim.2 = 0.458), ant genera (Dim.1 = 0.782, Dim.2 = 0.725), and plant family (Dim.1 = 0.784, Dim.2 = 0.723). Interestingly, both predation and parasitism are positive along Dimension 1, while dispersal is negative for Dimension 1. Furthermore, both dispersal and parasitism are positive along Dimension 2, while predation is negative on this same dimension. A large grouping of observations was associated with predation, but the other large grouping separates dispersal and parasitism.

When the distribution of the MCA scores for ant genera was compared to collected data on primary ant diet, several trends emerged. Granivorous ants such as *Veromessor* and *Pogonomyrmex* were found near predation, while ants considered to be seed dispersers like *Formica* were near dispersal. However, some ants generally considered to be granivores (e.g., *Messor*) exhibited both dispersal and predation interactions according to the MCA and when we checked the dataset. Genera with more variable feeding habits such as *Pheidole* and *Solenopsis* were located between dispersal and parasitism, or between predation and parasitism. Interestingly, genera known to be fungus‐farmers (*Acromyrmex*) were placed between parasitism and predation.

## 4 DISCUSSION

Our analysis of over 6,000 pairs of ant–seed interactions worldwide reveals predictable outcomes of the interactions based on ant and plant traits, habitat types, and the taxonomic associations of ants and plants that occur across several continents. Both the generalized linear models of traits, classification tree, and the multiple correspondence analysis showed that the outcome of ant–seed interaction varies continuously from dispersal to predation, but that parasitism involved a slightly different suite of ant and seed traits to explain its occurrence. These interactions result in outcomes falling along a gradient of benefits and costs received by the plant from predation to mutualism.

### 4.1 Seed size and morphology

Seed size was a key predictor of seed dispersal and predation. Small seeds (<1 mm) were much more likely to be consumed rather than dispersed, whereas predation and dispersal outcomes were almost equally likely among plants with larger seeds (Figure 2). Aside from size, all three outcomes of ant–seed interactions also depended on seed morphology including characteristics other than having an elaiosome. Seed shape was an important component of all outcome models and even exhibited an interaction effect in the dispersal model. Several studies have indicated that ants will readily use an elaiosome as a “handle” when transporting the seed (Beattie, Culver, & Pudlo, 1979; Byrne & Levey, 1993; Hughes & Westoby, 1992; Pulliam & Brand, 1975). Our analysis showed that long and narrow seeds (often shaped for wind or vertebrate dispersal) without elaiosomes were more readily dispersed, whereas spherical seeds were negatively associated with dispersal. Furthermore, the probability of parasitism increased with spherical seeds, which typically were larger and had no “handle” for ants. Seed morphology might dictate seed attractiveness to ants regardless of other incentives (elaiosomes) based on the ergonomics of carrying seeds. Seed traits were sometimes correlated with the plant growth habit and life‐form. The affinity of ants for easily handled seed structures might explain, in part, why ant dispersal was positively associated with grasses which tended to have long/narrow seeds.

The contribution of ant size to outcome probability indicates a potential trade‐off in the nutritional content of the seed with the energy and ability required of the ant to move the seed (Crist & MacMahon, 1992; Kaspari, 1996; Ness, Bronstein, Andersen, & Holland, 2004). Very small ants (<1.0 mm) will not have the energy or the physical ability to move large seeds, and so can only use a seed resource if a component of the seed is removed on the spot (Edwards, Hassall, Sutherland, & Yu, 2006). Parasitism might be a more accessible way to utilize such seed resources as granivory requires more specialized adaptations for seed removal and consumption (Lundgren, 2009). As we had relatively few studies that recorded parasitism, more targeted research needs to be completed on both the reasons and mechanisms ants parasitize seeds to determine if this is consistently true. Correlations between shape, size, and plant growth habit in conjunction with energy × nutrient trade‐offs might also contribute to the ant preferences and final seed interaction outcomes. This is exhibited by a greater probability of predation for seeds from grasses (long/narrow) and woody plants (odd and round/flat) which tended to be both larger and potentially easier to carry. A similar trend was seen in a field study of seed traits that examined the likelihood of seed removal from the seed bank—longer, heavier seeds were more likely to be taken (Traba, Azcarate, & Peco, 2006). Our data also suggest that dispersal outcomes exhibit the opposite trend—with smaller seeds of forbs more likely to be dispersed. Large amounts of variation in seed size (such as that seen by the grasses in our dataset) might help explain probabilities both dispersal and predation events, indicating that the growth habits of plants are not perfect indicators of seed size and should be considered in conjunction with other aspects of seed morphology.

### 4.2 Habitat and dietary variation in ant–seed interactions

Ant diet, particularly the consumption of plants (granivory and fungus‐farming), was correlated with all seed outcomes in the linear models. Seed preferences and outcomes might be dictated, in part, by seasonal or age‐related dietary requirements of the ant colonies such as preparing for a food drought/pulse and colony expansion (Mooney & Tillberg, 2005). For instance, peak foraging times for *Formica lugubris* Zetterstedt and *Camponotus cruentatus* Latreille have been shown to correspond to the seed maturation of the ant‐dispersed plant *Helleborus foetidus* L. (Boulay, Carro, Soriguer, & Cerda, 2007). Harvester ant studies indicate that temporal changes in dietary selection can cause ants to be less choosy about their seed selections and influences the foraging distance (Belchior et al., 2012; Mehlhop & Scott, 1983; Rissing, 1988; Whitford, 1978). In addition, *Solenopsis invicta* Buren, *Pheidole megacephala* (F.), and *Ochetellus glaber* (Mayr) have been shown to alter their foraging patterns to reflect a preference for protein‐rich foods during times of increased brood production (Cornelius & Grace, 1997; Stein, Thorvilson, & Johnson, 1990).

In addition to the shifting dietary requirements of ant colonies, the habitat surrounding ant–seed interactions could have influenced the seed outcome. As expected based on the prevalence of ant‐dispersed ephemerals, forest systems exhibited higher levels of seed dispersal (Handel & Beattie, 1990; Handel, Fisch, & Schatz, 1981). Seed predation was positively associated with arid environments like desert and grasslands which tend to be associated with granivorous ant species (Albrecht & Gotelli, 2001; Davidson, 1977; Whitford, Van Zee, Nash, Smith, & Herrick, 1999). However, sometimes the seed outcome was dependent on the interactions of habitat and ant diet and the growth habit of the plant. For instance, the ant genus *Messor* was observed dispersing grass seeds in forest and crop habits but predated grasses in grasslands. The same trend held true for *Messor* interactions with forb/herbs in forested and human habitats and woody plants in shrublands. Such variation in outcomes might be indicative of the resource constraint experienced by ants within different habitats (Clare, Barber, Sweeney, Hebert, & Fenton, 2011). For example, *Pogonomyrmex* harvester ants in North America will gather suboptimal seeds when the seed supply is low but later discard them once high‐quality seeds are found in the environment (Crist & MacMahon, 1992). Seeds might be more protected from predation in environments with more abundant food sources regardless of predator abundance, with other food resources diluting the risk to any given seed. Therefore, habitat interactions with dietary preferences could be based on local community dynamics of plants and the resultant vegetative structural variation (Christianini & Galetti, 2007; Rey et al., 2002).

### 4.3 Ant–seed interactions occur along a gradient of potential benefit and cost to plants

The range of outcomes in plant–ant interactions shown here is not dissimilar to the range of outcomes documented in other plant–mutualist interactions. Johnson et al. (1997) observed the range of interactions between plant and their mycorrhizae, where interactions ranged between mutualism and antagonism with mycorrhizal parasitism of the plant in between. Given results from generalized linear models showing opposite trends between dispersal and predation, we anticipated that dispersal and predation would occur along a single gradient. When using classification trees and MCA, we found that our predictions about the gradient of species interactions from myrmecochory (dispersal) to granivory (predation) were correct as there was a gradient from dispersal to predation, but with parasitism more highly associated with predation in its occurrence among habitats and ant or seed traits. The placement of players along this gradient did not necessarily follow expected patterns. While some ants with well‐studied dietary preferences were placed similarly to their previously assumed position on the MCA (i.e., *Veromessor* and predation, *Formica* and dispersal) (Feener & Lighton, 1991; Gorb & Gorb, 1995, 1999; Tevis, 1958), a few were found to contribute to multiple seed outcomes. The ant genus *Messor* (granivores) was found between predation and dispersal and *Acromyrmex* (fungus‐farmers) between predation and parasitism, indicating that not all feeding habits are static and result in interactions located between the extremes (Azcarate & Peco, 2007; Retana, Pico, & Rodrigo, 2004; Rockwood & Hubbell, 1987).

Although intermediate cases between mutualistic and antagonistic interactions have not been well studied for any plant mutualism, the literature indicates they do exist and come with their own arms races. Ant‐mediated plant protection from herbivores has been well documented in many ant and plant species where the plant provides food and shelter in return for an aggressive ant (Gaume, McKey, & Anstett, 1997; Janzen, 1969). This mutualism can devolve into the ant using the plant without providing services and interfering with other mutualisms (i.e., pollination) or the plant removing the incentives but still receiving protections (Guimarães, Rico‐Gray, dos Reis, & Thompson, 2006; Ness, 2006; Palmer et al., 2008). The mutualism can also degrade in more subtle ways when the ants trim off plant inflorescences and plants, in turn, reduce the amount of shelter provided to the ants (Izzo & Vasconcelos, 2002). Our data build upon the prior work in other plant systems that have shown a continuous range of variation in the outcome of species interactions that vary by habitats or the taxonomic identities of the participants. Mycorrhizae are more likely to be detrimental in simplified systems and ant bodyguards are less likely to work for the plant's benefit when herbivory is minimal or incentives are offered by a competitor (Johnson et al., 1997; Renault, Buffa, & Delfino, 2005; Szentesi & Schmera, 2011). These contexts may be determined not only by nutritional requirements but also in terms habitat and the relative proportion of certain resources made available by the plants present (Dejean, Bourgoin, & Gibernau, 1997; Sanders & Gordon, 2000).

Perhaps not surprisingly, the relationships between continent and seed outcome with the placement of plant families on the MCA showed that continent and taxonomic identity were associated. The plant family Arecaceae (palm trees) was placed near parasitism on the MCA and was found in South America, the continent with the greatest probability of parasitism. One of the plant families near dispersal on the MCA, Cistaceae, was also found often in Europe, the continent with the highest probability of dispersal. Poaceae was placed in between dispersal and predation on the MCA (potentially due to the variation in seed sizes and shapes) and was also most commonly observed in conjunction with Europe, Asia, and Africa. In terms of seed outcomes, Asia and Africa appear more similar to each other than Europe, adding an additional layer of variation to seed outcomes experienced by Poaceae. The relationships of plant family to continent may be due, in part, to phylogenetic relatedness, as continents tend to house certain habitats that then influence both the biotic and abiotic conditions influencing plant traits (Eiserhardt, Svenning, Kissling, & Balslev, 2011; Fernández‐Mazuecos & Vargas, 2010, 2011). This potential influence of phylogenetic relatedness on shared traits such as seed shape and ant size across spatial scales could be used for better predictions species interactions outcomes in the future (Beck & Kitching, 2007; Graham & Fine, 2008; Losos, 2008).

### 4.4 Gaps in ant–seed interaction knowledge

Our review is based on an unprecedented number of studies involving ant–seed interactions, allowing us to assess knowledge gaps and future directions. There was an unequal representation of studies among geographic locations, habitat types, and the ant and plant taxa (Clark & Wilson, 2003; Forget & Wenny, 2005; Lambert, Hulme, & Wall, 2005), potentially limiting our ability to separate different sources of variation in the outcomes of interactions between ants and seeds. Our observed seed outcomes in human environments (i.e., crops and urban areas) were skewed toward studies in arid environments (Australia, Portugal, Spain, and southwestern USA) where granivorous ants tend to be pests (Diaz, 1992, 1994), leaving us with little information about how ants react in human ecosystems while facing different abiotic constraints. Furthermore, even within well‐studied systems, we cannot perfectly predict ant–seed interactions because little is still known on the impacts of accidental dispersal and the specifics of ant diets at either genus or species level (AntWiki, 2017; Coovert, 2005). With the exception of granivores, fungus‐farmers, and some pest species, most dietary information appears to be gathered anecdotally or from artificial food sources at baits (Albrecht & Gotelli, 2001; Carroll & Janzen, 1973; Cassill & Tschinkel, 1998; Cerda, Retana, Carpintero, & Cros, 1996; Hooper‐bùi, Appel, & Rust, 2002; Rockwood & Hubbell, 1987; Véle & Modlinger, 2017).

Determining the outcome of ant–seed interactions in the field is not always straightforward and many studies focus on either seed predation or dispersal with parasitism observed occasionally. Therefore, one of the limitations of our synthesis is that true rates of both dispersal and predation are rarely measured simultaneously (Byrne & Levey, 1993; Vander Wall, Kuhn, & Beck, 2005). A strict focus on dispersal and predation might mask the occurrence of more nuanced interactions that fall into parasitism or amensalism and commensalism. Lastly, the majority of our data were derived from the assumption that the stated seed outcome was the actual fate of the seed; but as it is relatively difficult to follow seeds from seed set to germination, many of the studies used in this review did not determine ultimate seed fate (Andersen, 1989; Christianini et al., 2007). For instance, granivorous ant species such as *Pogonomyrmex* have been shown to be rather sloppy foragers (Arnan, Rodrigo, Molowny‐Horas, & Retana, 2010; Bullock, 1974; Detrain & Tasse, 2000). The dropped or neglected seeds are then given a dispersal boost without the normal predation risk, or they could be brought into the nest for consumption but remain for too long and germinate within the nest (Aranda‐Rickert & Fracchia, 2011; Mull, 2003; Retana et al., 2004). Although difficult, following the ultimate fate of the seed would account for other indirect effects ants have on those seeds, allowing more accurate assessment of the interaction along the gradient of plant benefit. We have also assumed that ant species are relatively homogenous within a genus although this is truer of select genera. For instance, the ant genera *Pogonomyrmex* and *Veromessor* are well studied, and species within those genera appear to inhabit similar trophic niches. A genus like *Pheidole* is very species‐rich and diverse in terms of niches (some are predators, while others are omnivorous), making predictions for genus‐level seed interactions less accurate (Armbrecht, Perfecto, & Vandermeer, 2004; Bernstein, 1979; Economo et al., 2015). Due to this variation and with the addition of more precise dietary information, use of functional foraging groups might be a better way to predict the ant side of these seed interactions. Despite these limitations, our global review suggests that the likely outcomes of ant–seed interactions can be predicted in a general way from ant and plant traits, habitat types, and taxonomic identities. These general properties should provide a framework for more specific predictions of outcomes in particular regions, ant–plant associations, or management regimes.

## CONFLICT OF INTEREST

None declared.

## AUTHOR CONTRIBUTIONS

HJP collected and input reference data, performed statistical analyses, and contributed to the writing and revising of the manuscript. TOC informed the direction of the manuscript, performed statistical analyses, and contributed to the writing and revising of the manuscript.

## DATA ACCESSIBILITY

All data and source reference information from this manuscript will be available online as supplemental materials.
